# The relationship between weight change and risk of hip fracture: meta-analysis of prospective studies

**DOI:** 10.1038/srep16030

**Published:** 2015-11-02

**Authors:** Qing-Bo Lv, Xin Fu, Hai-Ming Jin, Hai-Chao Xu, Zhe-Yu Huang, Hua-Zi Xu, Yong-Long Chi, Ai-Min Wu

**Affiliations:** 1 Department of Orthopaedics, Second Affiliated Hospital of Wenzhou Medical University, Second Medical College of Wenzhou Medical University, Wenzhou, Zhejiang, China; 2 Department of Orthopaedics, Tianjin Hospital, Tianjin, China

## Abstract

The relationship between weight change and risk of hip fracture is still controversial. We searched PubMed and Embase for studies on weight change and risk of hip fracture. Eight prospective studies were included. The weight loss studies included 85592 participants with 1374 hip fractures, and the weight gain studies included 80768 participants with 732 hip fractures. Weight loss is more likely a risk factor of hip fracture, with an adjusted RR (Relative Risk) (95% CI) of 1.84 (1.45, 2.33). In contrast, weight gain can decrease the risk of hip fracture, with an adjusted RR (95% CI) of 0.73 (0.61, 0.89). Dose-response meta-analysis shows that the risk of hip fracture is an ascending curve, with an increase of weight loss above the line of RR = 1; this trend is consistent with the results of forest plots that examine weight loss and hip fracture. For weight gain and risk of hip fracture, the descending curve below the line of RR = 1; this trend is consistent with the results of forest plots that examine weight gain and hip fracture. Our meta-analysis suggests that weight loss may be a risk factor for hip fracture and that weight gain may be a protective factor for hip fracture.

Fracture has increasingly become a main cause of morbidity in the population as well as a significant global health concern^1^,^2^. As the ageing population increases, fracture is predicted to increase in the future^3^,^4^,^5^, with studies suggesting that the number of hip fractures in the world will increase to 6.26 million by 2050. To decrease the incidence of fracture, medical personnel need to be knowledgeable of its risk factors and protective factors^6^,^7^,^8^.

There are many factors that may promote the risk of or protection from fracture, such as vitamin D, vitamin A and smoking status^9^,^10^,^11^. Whether weight change is a risk or a protective factor for hip fracture is an important and still controversial issue^12^,^13^,^14^,^15^.

Langlois *et al.*^12^ followed 2413 white men (8-year follow-up with 13620 person-years) and found that the participants who experienced more than 10% weight loss had an increased risk of hip fracture but that those who experienced more than 10% weight gain had protection against the risk of hip fracture. Researchers supposed that weight loss may reduce mechanical loading, which is important to stimulate bone formation and increase bone mineral density^16^. Poor health and muscle loss associated with weight loss may also increase hip fracture risk in people who lose weight^17^. Similar results indicating that weight loss increases the likelihood of hip fracture were reported by Ensrud *et al.*^18^.

However, in the study conducted by Meyer *et al.*^14^, the weight loss group did not show a significant increase in hip fracture risk compared with the weight unchanged group. In contrast, French *et al.*^15^ reported that weight gain did not significantly increase or decrease the risk of hip fracture, with an RR of 0.96 (0.59, 1,57). Similar results were reported in the study by Langlois *et al.*^13^, who found that weight gain did not significantly decrease the risk of hip fracture; in their study, the samples were separated into three groups according to BMI (BMI < 23.0; 25.9 > BMI > 23.0; BMI > 26.0). Their results showed that none of the groups exhibited a significant decrease in the risk of hip fracture, with RR = 1.2 (0.6, 2.4); 0.8 (0.3, 1.8); and 0.6 (0.2, 1.9), respectively.

Because the results reported by previous studies were inconsistent, the purpose of the meta-analysis that we conducted was to determine the relationship between weight gain/loss and the risk of hip fracture based on current evidence from prospective studies.

## Methods

The present study accords with the preferred reporting items for systematic review and meta-analyses (PRISMA) guidelines (**Checklist S1**).

### Search strategy

We conducted a literature search in PubMed and Embase on Oct. 16, 2014 using the following keywords: 1) weight variability, or weight change, or weight loss, or weight gain; 2) hip fractures or hip fracture; and 3) cohort study, cohort studies, prospective study, or prospective studies, or longitudinal study. The “related article” function was also used during the search; the references for retrieved articles were manually searched to avoid initial misses.

### Selection criteria

We included studies in this meta-analysis based on the following criteria: (1) Designed as a prospective cohort study; (2) exposure of interest included either weight variability, weight change, weight loss, or weight gain; (3) primary outcome of interest was hip fracture; and (4) relative risk (RR) estimates with 95% CI (confidence interval) were reported or could be calculated using the reported data. If multiple studies used the same dataset, only the one with more detailed or updated data was included in this meta-analysis. All potential studies were reviewed independently for eligibility by two authors; disagreements were discussed and resolved with a third independent author.

### Data extraction

Two authors independently evaluated all of the relevant articles to identify eligible studies from the PubMed and Embase databases; the third author checked for consistency between the authors. We also extracted the study characteristics for each trial, and the following data were recorded: first author’s last name; publication year; country in which the study was performed; study period; sample size (cases and controls or cohort size); gender and age of participants; measure and range of exposure; variables adjusted for analysis; and RR estimates with corresponding 95% CI for each category of weight variability, weight change, weight loss, or weight gain levels. If there were two or more RRs of different potential confounders, we extracted the RRs that reflected the greatest degree of control for potential confounders. We contacted authors of the primary studies for additional information when necessary. The study quality was assessed by using the nine-star Newcastle-Ottawa Scale^19^.

### Statistical analysis

We used RR and combined the estimates using a random-effects model^20^,^21^,^22^ with the 95% CI to obtain a common measure of the relationship between weight change and the risk of hip fracture across studies. To obtain more accurate results, the RRs and 95% CIs of both the largest loss and the largest gain vs. the least changes RR category were separately pooled for synthesis.

We also conducted a meta-analysis examining the potential nonlinear dose-response between weight loss/gain and risk of hip fractures using restricted cubic splines with four knots at percentiles 5%, 35%, 65% and 95% of the distribution^23^. The dose-response meta-analysis was conducted according to the random-effects meta regression model, as reported by Greenland and Longnecker and Orsini *et al.*^24^,^25^. To make the included studies consistent, all of the percentage values (%) were calculated as weight values (Kg).

Statistical heterogeneity was evaluated using the Q (χ^2^) and I^2^ statistics^26^; p > 0.10 and I^2^ < 30% were defined as not having significant heterogeneity. Sensitivity analysis was evaluated by determining whether the remaining results would be markedly affected after removing one study. To conduct the sensitivity analysis, each study involved in the meta-analysis was singly deleted to determine the influence of the individual data set on the pooled RRs after each removal. Potential publication bias was evaluated using the Egger regression asymmetry test^27^. All statistical tests were performed using STATA software (version 12.0; Stata Corp, College Station, TX, USA).

## Results

### Literature search

**Figure 1:**
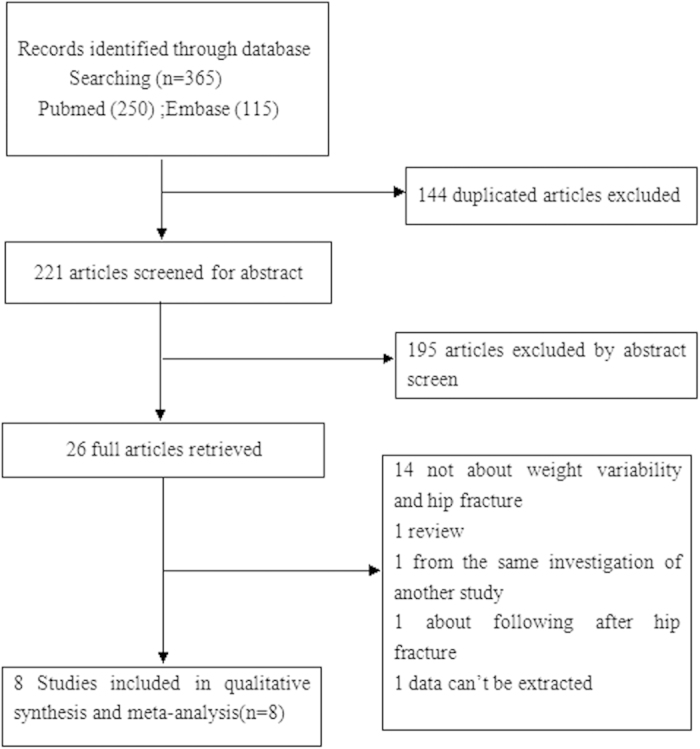
The selection of literature for included studies.

The method used to select the studies included in this meta-analysis is shown in Fig. 1. Three hundred sixty-five potential records were identified from the databases, including one hundred forty-four duplicated articles. One hundred ninety-five papers were excluded by abstract screen, and twenty-six full articles were retrieved. Fourteen articles were excluded because they were not related to weight change and hip fracture; one was a review, one was from the same site as another included study, one examined patients after hip fracture, and the data could not be extracted from another. Finally, five weight change studies^12^,^13^,^14^,^15^,^28^ and three weight loss only studies^18^,^29^,^30^ were included in this meta-analysis. Meyer *et al.*^31^ published a study in 1995 that was updated in 1998^14^; therefore, only the 1998 article was included.

### Study characteristics

The characteristics of the included weight loss studies are shown in Table 1, and the weight gain studies are shown in Table 2. The weight loss studies included 85592 participants with 1374 hip fracture cases, and the weight gain studies included 80768 participants with 732 hip fracture cases. The detailed results of study quality based on the nine-star Newcastle-Ottawa Scale are shown in Table 3.

### Weight change and hip fracture risk

**Figure 2:**
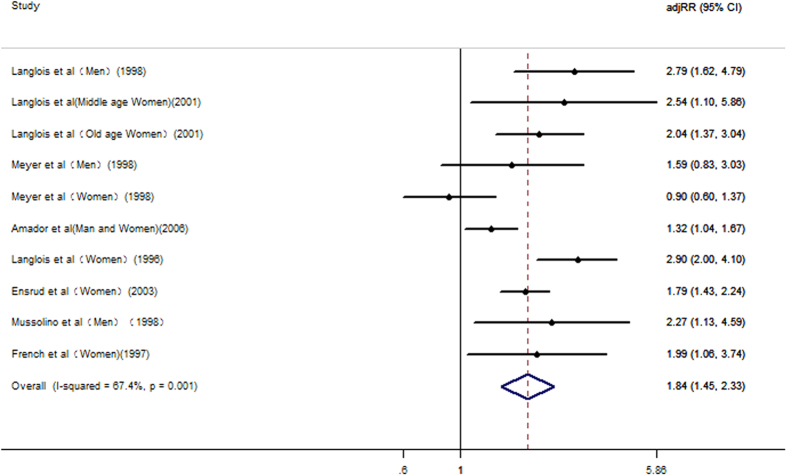
Adjusted Relative Risks of hip fracture for the highest *vs.* reference category of weight loss.

**Figure 3:**
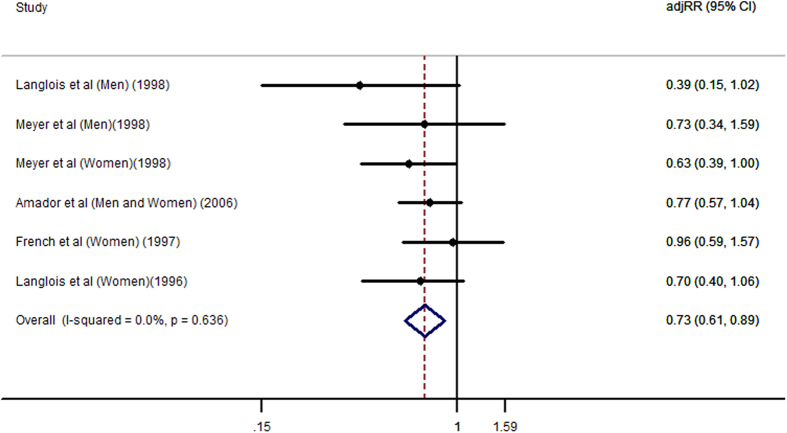
Adjusted Relative Risks of hip fracture for the highest *vs.* reference category of weight gain.

Eight studies^12^,^13^,^14^,^15^,^18^,^28^,^29^,^30^ examined the relationship between weight loss and risk of hip fracture. In addition, five studies^12^,^13^,^14^,^15^,^28^ evaluated the relationship between weight gain and risk of hip fracture. The results of the meta-analysis are shown in Figs 2 and 3. The results from the present meta-analysis for the highest versus reference category showed that the adjusted relative risks (RR) of weight loss and weight gain related to hip fracture were 1.84 (1.45, 2.33) and 0.73 (0.61, 0.89), respectively. Statistically significant evidence of heterogeneity was found in weight loss studies (I^2^ = 67.4%, *P* = 0.001) but not in weight gain studies (I^2^ = 0.0%, *P* = 0.636). A sensitivity analysis of weight loss found that there was no significant change when any one study was omitted. Egger’s test showed there was no significant publication bias, with *P* =  0.317 for weight loss studies and *P* = 0.294 for weight gain studies.

### Dose-response meta-analysis

**Figure 4:**
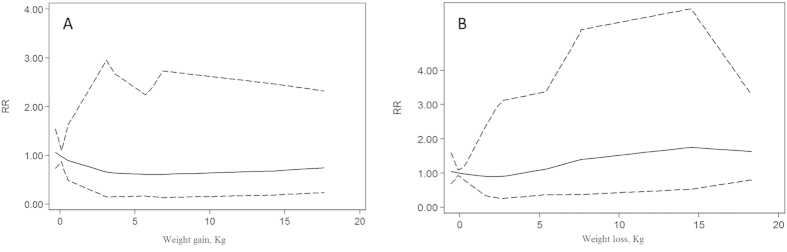
Dose-response relationship between weight gain (A) and weight loss (B) and relative risk of hip fracture. The solid line represents adjusted relative risk, and dotted lines represent the 95% confidence intervals for the fitted trend. The adjusted RR of weight gain is a descending curve below the line of RR = 1, whereas the adjusted RR of weight loss is an ascending curve above the line of RR = 1. The line trend of dose-response meta-analysis is consistent with the results of the forest plots in Figs 2 and 3.

Only four^12^,^14^,^28^,^29^ of the eight studies on weight loss and risk of hip fracture and three^12^,^14^,^28^ of the five studies on weight gain and risk of hip fracture met the dose-response meta-analysis criteria (Fig. 4). The result of generalised least-squares regression for weight gain and the adjusted RR of hip fracture was a descending curve below the line of RR = 1 (Fig. 4A); for weight loss and the adjusted RR of hip fracture, it was an ascending curve above the line of RR = 1 (Fig. 4B). The line trend of the dose response meta-analysis was consistent with the results of the forest plots shown in Figs 2 and 3.

## Discussion

Many studies have been conducted to evaluate the risk factors for fracture due to the increased prevalence of fracture. Low body mass index (BMI) has been proven to be a risk factor for fracture, especially fracture of the hip^32^,^33^. Moreover, obesity has been reported to decrease the risk of hip fracture^34^. Other factors such as smoking and the vitamin D and vitamin A levels have also been proven to influence the risk of fracture^9^,^10^,^11^. Weight change (including weight gain and weight loss) has been considered an important factor influencing the risk of hip fracture^12^,^13^,^14^,^15^,^28^,^29^,^30^. We conducted this meta-analysis to systematically evaluate the relationship between weight change and risk of hip fracture.

Our meta-analysis shows that weight loss can increase the risk of hip fracture. Compared with the reference group of people who maintain a stable weight, those who experience weight loss are more likely to develop hip fracture, with an adjusted RR = 1.84, 95% CI (1.45, 2.33). There are several reasons why weight loss can cause bone loss and increase the risk of hip fracture. People who experience weight loss, whether voluntary or involuntary, may decrease their intake of the nutrients that are required to maintain bone mineral density, such as protein, calcium, and vitamin D^35^; a deficiency in these may contribute to bone loss^36^. Furthermore, the decreased strain on the bones imposed by lower body mass due to weight loss may lead to decreased bone mineral density and impaired structural integrity of the bones^34^. In addition, people with involuntary weight loss may have more indicators of impaired health, which may be both the cause of weight loss and a risk for both decreased bone mineral density and increased falls and fracture^37^,^38^,^39^. Finally, according to some studies, weight loss may be connected with hormone levels, including the reduction of growth hormone^40^, increases of serum glucocorticoids^41^, and an unbalanced disturbance of central and peripheral leptin levels^42^. These changes can cause the observed weight loss and lower bone mineral density.

Statistically significant evidence of heterogeneity was found among the weight loss studies (I^2^ = 67.4%, P = 0.001), and the sensitivity analysis found no significant change after any study was omitted (Figure S1). Differences in study participants’ race, participants’ BMI and follow-up duration among the studies may be the source of heterogeneity. Therefore, because heterogeneity cannot be eliminated in this study, we suggest that this result should be interpreted with caution.

Compared with weight loss, weight gain may contribute to increased bone mineral density and a decreased risk of hip fracture. Three factors may induce increased bone mineral density: increased adipose-based production of oestrogens, increased gravitational force on bone, and decreased likelihood of injury in the event of a fall. Specifically, falling injury is less likely because the increased protective padding around the hip can decrease the impact force to the joint^43^,^44^. Additionally, some people who gain weight may have a lower level of physical activity, thus reducing their exposure to the risk of falling^12^. However, people with weight gain may exhibit a heavy body, which is associated with the presence of medical conditions including physical disability^45^, a low mental status score, a low level of physical activity, and current tobacco use^46^,^47^. Increased weight may also contribute to an increased risk of hip fracture; therefore, the assignment of weight gain as a protective or risk factor for hip fracture is inconsistent. This meta-analysis shows that weight gain can decrease the risk of hip fracture, with an adjusted RR = 0.73, 95%; CI: 0.61 to 0.89; no significant heterogeneity was observed. Although most of the included studies did not show a significant difference, the pooled analysis revealed that weight gain can decrease the risk of hip fracture by approximately 27%. The result of the dose-response analysis is a descending curve below the line of RR = 1. The line trends of the dose-response meta-analysis for both weight gain and weight loss are consistent with the results of the forest plots.

### Strengths of this study

To the best of our knowledge, this is the first systematic review and meta-analysis of the relationship between weight variability and risk of hip fracture in prospective studies. In this article, we carried out an extensive quality assessment and investigated heterogeneity with sensitivity analyses. Our quantitative assessment is based on prospective studies, which overcome the shortcomings of recall or selection bias in retrospective studies. In addition, study-specific RR estimates were combined using a random-effects model, which considers both within- and between-study variations^21^. Moreover, the long duration of follow-up and the large number of participants are two additional strengths of this meta-analysis.

### Limitations of this study

There are several limitations to this meta-analysis. First, we did not perform a subgroup analysis by gender and age. Additionally, the “pre-study weight” (overweight or non-overweight) and the willingness to “try to lose weight” (intentional versus unintentional weight loss) may influence the risk of fracture. Ensrud *et al.*^18^ reported that if the BMI < 25.9 kg/m^2^, no significant difference was found in the intentional weight loss group, with a RR of hip fracture of 2.00 (0.83–4.80). However, a significant difference was found in the unintentional weight loss group, within which the RR of hip fracture was 1.47 (1.11, 1.94). If the BMI > 25.9 kg/m^2^, both the intentional and unintentional weight loss groups had a significantly different RRs: 2.48 (1.33, 4.62) and 2.45 (1.49, 4.04), respectively. Therefore, it could be suggested that intentional weight loss in the non-overweight population (BMI < 25.9 kg/m^2^) will not increase the risk of hip fracture, while unintentional weight loss in the non-overweight population (BMI < 25.9 kg/m^2^) and both intentional and unintentional weight loss in the overweight population (BMI > 25.9 kg/m^2^) will increase the risk of hip fracture. Unfortunately, only one study provided RR data for intentional and unintentional weight loss and the risk of hip fracture; therefore, a meta-analysis could not be performed. Furthermore, we could not estimate some of the confounding factors that were inherent in the included studies. Statistically significant evidence of heterogeneity was found in weight loss studies, though we performed a sensitivity analysis of weight loss and found there was little change to the direction of effect when any one study was excluded. Moreover, due to a lack of critical data, not all of the studies met the requirement for the dose-response meta-analysis. Finally, the measurement of weight change in most previous studies did not consider relative fat and lean mass change, though the gain in fat mass may have a negative effect on BMD, thus further influencing the risk of hip fracture. Therefore, this could be a direction for future research.

## Conclusion

This meta-analysis of prospective studies finds that weight loss may increase the risk of hip fracture but that weight gain may decrease the risk of hip fracture.

## Additional Information

**How to cite this article**: Lv, Q.-B. *et al.* The relationship between weight change and risk of hip fracture: meta-analysis of prospective studies. *Sci. Rep.* **5**, 16030; doi: 10.1038/srep16030 (2015).

## Supplementary Material

Supplementary Information

## Figures and Tables

**Table 1:** Characteristics of Prospective Studies on Weight Loss and Hip Fracture.

Source	No. of participants	Location/Period	Gender	Age (years)	No. of cases^a^	Measure/Range of Loss	Study Quality^b^	Adjustment for Covariates^c^
Langlois *et al.* 1998	2413	United States 1985–1992	M	67–104	72 HF	Weight Loss: Q1≤5% 5% < Q2 < 10% Q3≥10%	7	BMI at age 50 year, age, number of medical conditions, low mental status score, physical disability.
Langlois*et al.* 2001	2180	United States 1982–1992	F	50–74	171 HF	Weight Loss: Q1≤5% 5% < Q2 < 10% Q3≥10%	8	BMI, age at baseline; cigarette smoking (current, former, never); history of chronic diseases based on self-reported doctors’ diagnoses of bronchitis, thyroid disease, diabetes, kidney disease, heart disease or stroke; and alcohol consumption (none or any consumed in the past year).
Meyer *et al.* 1998	39089	Norway 1974–1978	F:19938 M:19151	37–58	207HF	Weight Loss (kg/12 years): F:Q1:Loss of 1.3 to gain of 1.5 Q2:Loss of >1.3 M:Q1:Loss of 0.9 to gain of 2.0 Q2:Loss of >0.9	9	Age at screening, weight variability (root mean square error), mean body mass index, body height, self-reported physical activity at work and during leisure, diabetes mellitus, disability pension, marital status and smoking habits.
Amador *et al.* 2006	1749	United States 1993–2001	F: 1008 M: 741	≥65	18HF	Weight Loss: Q1≤10% Q2 > 10%	7	Sociodemographic variables included age and gender, smoking status, medical conditions, depressed symptomatology, BMI, waist circumference, grip strength.
Langlois *et al.* 1996	3683	United States 1983–1992	F	67–104	253HF	Weight Loss: Q1≤5% 5% < Q2 < 10% Q3≥10%	8	Age at baseline, body mass index at age 50 years, cigarette smoking, alcoholconsumption in the past year, number of medical conditions, impaired mobility, and use of thiazide diuretics.
Ensrud *et al.* 2003	6785	United States 1986–2001	F	≥65	400HF	Weight Loss: Q1<5% Q2≥5%	8	Age, health status, smoking, physical activity, medical conditions, history of fracture, BMI, neuromuscular function, and hipbone density.
Mussolino *et al.* 1998	2879	United States 1982–1992	M	45–74	71HF	Weight Loss: Q1≤5% 5%<Q2<10% Q3≥10%	8	Self-reported data on age at baseline, previous fractures other than hip (none, any), smoking status (current, not current), alcohol consumption in the past year (none, any), nonrecreational physical activity (much, moderate, little or no exercise), chronic disease prevalence, calcium intake (mg/day), calories (kcal/day), protein consumption (g/day), and weight loss from maximum.
French *et al.* 1996	33834	United States 1986–1992	F	55–69	182HF	Weight loss: Q1≤10% Q2>10%	7	Baseline values of age, waist/hip ratio, BMI, BMI^2^, smoking status (never, former, current), pack years of cigarettes, education (<high school, high school, >high school), physical activity (low, medium, high), alcohol (0, <4, ≥4 g/d), marital status (yes/no), hormone replacement (never, former, current).

^a^HF: Hip fracture.

^b^Study quality was judged based on the Newcastle-Ottawa Scale (range, 1–9 stars).

^c^BMI: body mass index; BMD: Bone mineral density.

**Table 2:** Characteristics of Prospective Studies on Weight Gain and Hip Fracture.

Source	No. of participants	Location/Period	Gender	Age (years)	No. of cases^a^	Measure/Range of Loss	Study Quality^b^	Adjustment for Covariates^c^
Langlois *et al.* 1998	2413	United States 1985–1986	M	67–104	72 HF	Weight Gain: Q1≤5% 5%<Q2<10% Q3≥10%	7	BMI at age 50 year, number of medical conditions, low mental status score, physical disability.
Meyer *et al.* 1998	39089	Norway 1974–1978	F:19938 M:19151	37–58	207HF	Weight Gain(kg/12 years): F:Q1: Loss of 1.3 to gain of 1.5 Q2: Gain of 1.6 to 4.6 Q3:Gain of ≥ 4.7 M:Q1: Loss of 0.9 to gain of 2.0 Q2: Gain of 2.1 to 5.2 Q3:Gain of ≥5.3	9	Age at screening, weight variability (root mean square error), mean body mass index, body height, self-reported physical activity at work and during leisure, diabetes mellitus, disability pension, marital status and smoking habits.
Amador *et al.* 2006	1749	United States 1993–2001	F: 1008 M: 741	≥65	18HF	Weight Gain: Q1≤10% Q2>10%	7	Sociodemographic variables included age and gender, smoking status, medical conditions, depressed symptomatology, BMI, waist circumference, grip strength.
French *et al.* 1996	33834	United States 1986–1992	F	55–69	182HF	Weight Gain: Q1<10% Q2>10%	7	Baseline values of age, waist/hip ratio, BMI, BMI^2^, smoking status (never, former, current), pack years of cigarettes, education (<high school, high school, >high school), physical activity (low, medium, high), alcohol (0, <4, ≥4 g/d), marital status (yes/no), hormone replacement (never, former, current).
Langlois *et al.* 1996	3683	United States 1983–1992	F	67–104	253HF	Weight Gain: Q1≤5% 5%<Q2<10% Q3≥10%	8	Age at baseline, body mass index at age 50 years, cigarette smoking, alcohol consumption in the past year, number of medical conditions, impaired mobility, and use of thiazide diuretics.

^a^HF: Hip fracture.

^b^Study quality was judged based on the Newcastle-Ottawa Scale (range, 1–9 stars).

^c^BMI: body mass index; BMD: bone mineral density.

**Table 3:** Assessment of quality of included studies on the use of Nine-Star Newcastle-Ottawa Scale.

	Selection					Outcome assessment			
Study (authors, year)	Representativeness of the exposed cohort	Selection of the nonexposed cohort	Ascertainment of exposure	Incident disease	Comparability	Assessment of outcome	Length of follow up	Adequacy of follow up	Score
Langlois *et al.* 1998	*	*	*	*	*	—	*	*	*******
Langlois *et al.* 2001	*	*	—	*	**	*	*	*	********
Meyer *et al.* 1998	*	*	*	*	**	*	*	*	*********
Amador *et al.* 2006	*	*	—	*	**	*	—	*	*******
Langlois *et al.* 1996	*	*	—	*	**	*	*	*	********
Ensrud *et al.* 2003	*	*	*	*	*	*	*	*	********
Mussolino*et al.* 1998	*	*	—	*	**	*	*	*	********
French *et al.* 1996	*	*	—	*	**	—	*	*	*******

Note One asterisk means one score, studies with more scores on behalf of higher quality.
